# Element-Specific Ultrafast Lattice Dynamics in Monolayer WSe_2_

**DOI:** 10.1021/acs.nanolett.4c03611

**Published:** 2024-10-21

**Authors:** Hyein Jung, Shuo Dong, Daniela Zahn, Thomas Vasileiadis, Helene Seiler, Robert Schneider, Steffen Michaelis de Vasconcellos, Victoria C. A. Taylor, Rudolf Bratschitsch, Ralph Ernstorfer, Yoav William Windsor

**Affiliations:** † Institute for Optics and Atomic Physics, Technical University Berlin, Strasse des 17, Juni 135, 10623 Berlin, Germany; ‡ Department of Physical Chemistry, Fritz Haber Institute of the Max Planck Society, Faradayweg 4-6, 14195 Berlin, Germany; § Institute of Physics and Center for Nanotechnology, University of Münster, Heisenbergstraße 11, 48149 Münster, Germany

**Keywords:** monolayer WSe_2_, ultrafast electron diffraction, element-specific, nonthermal phonons

## Abstract

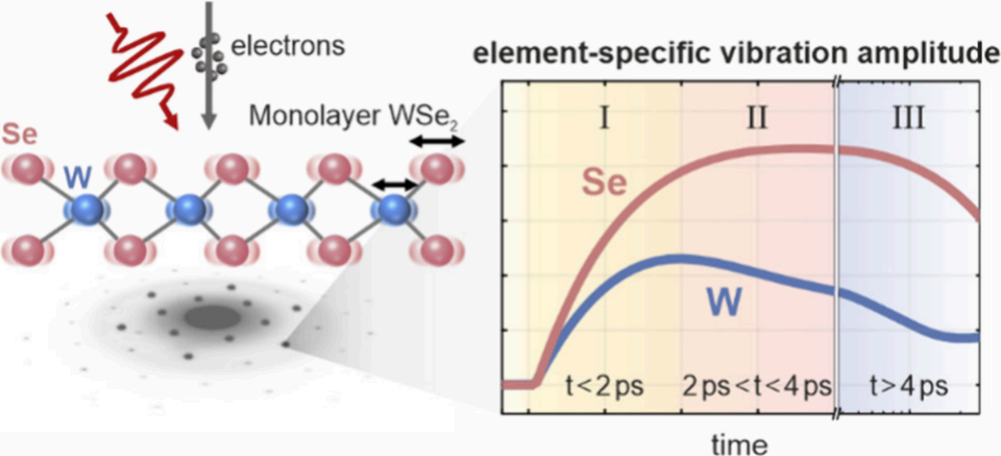

We study monolayer WSe_2_ using ultrafast electron diffraction. We introduce an approach to quantitatively extract atomic-site-specific information, providing an element-specific view of incoherent atomic vibrations following femtosecond excitation. Via differences between W and Se vibrations, we identify stages in the nonthermal evolution of the phonon population. Combined with a calculated phonon dispersion, this element specificity enables us to identify a long-lasting overpopulation of specific optical phonons and to interpret the stages as energy transfer processes between specific phonon groups. These results demonstrate the appeal of resolving element-specific vibrational information in the ultrafast time domain.

Van der Waals bonded materials consist of weakly bonded atomic layers, facilitating the practical synthesis of atomically thin crystalline layers. Such dimensional constraints can strongly alter material properties, such as indirect band gaps becoming direct.^1−3^ The availability of such monolayers fuels the realization of ultrathin crystalline monolayer-monolayer heterostructures, where the combination of functionally distinct monolayers enables new functionalities.^4−7^ Transition metal dichalcogenides (TMDCs) are such materials, which are predicted to play a central role in next-generation optoelectronics due to their infrared-to-visible bandgaps.^8−10^

For future applications, understanding the nonequilibrium behavior of monolayer TMDCs is paramount. To date, femtosecond studies have revealed a variety of nonequilibrium electronic phenomena.^11−16^ The ultrafast excitations in these studies are also expected to affect the lattice via electron–phonon coupling (EPC). EPC is expected to initiate a cascade of phonon–phonon scattering processes,^17,18^ through which the phonon population evolves to a new thermalized equilibrium state. Understanding subpicosecond phenomena within the lattice is of particular interest in monolayers due to their constrained dimensions, which have been recently shown to affect EPC.^19^

Beyond monolayer TMDCs, studying the subpicosecond evolution of phonons elucidates the coupling between vibrational modes, holding great potential for nanoscale devices, e.g. for engineering heat flow and novel control pathways.^20,21^ Despite this, experimental reports about the ultrafast evolution of phonon populations, in particular through nonthermal states (those that disobey Bose–Einstein statistics, meaning that temperatures cannot describe them), are rare. Femtosecond electron- and X-ray scattering techniques can access such information and have been reported on both metals^22−28^ and semiconductors.^29−33^ Ultrafast Bragg diffraction (elastic scattering), is particularly appealing because it provides quantitative real-space information about changes in the atoms’ arrangement and their vibrational amplitudes, including incoherent vibrations (via the Debye–Waller effect).^29,34^ Fully resolving such motions is particularly advantageous when studying multielement compounds, as it can elucidate different ions’ roles in the ultrafast response to photoexcitation. However, disentangling motions of different elements has proven to be challenging, and studies often resort to “effective” element-averaged information,^31,35−37^ or to comparison with theoretical simulations of such motion.^30,37,38^ In fact, to our knowledge, there are presently no reports in the literature in which crystal structures were fully resolved on subpicosecond time scales (apart from single elements).

A recent ultrafast electron diffraction (UED) study on NiO demonstrated progress toward element specificity^39^ by employing tabulated equilibrium Debye–Waller factors of each element and identifying photoinduced variations from them. Here we advance beyond this, and independently determine element-specific Debye–Waller factors (vibrational amplitudes) as functions of time, purely from the experimental data only. This provides a real space picture of incoherent atomic motion on sub-Å length scales and can highlight the role of individual atoms (this is complementary to the momentum resolution obtained by inelastic scattering^19,36,40−42^). For example, V-dominated incoherent vibrations around ∼3 THz were suggested as a key ingredient in the ultrafast structural phase transition in VO_2_.^38^

We present a UED study of monolayer WSe_2_. By recording hundreds of Bragg reflections, we disentangle and quantitatively determine the incoherent vibrational amplitudes of all W and Se atoms on femtosecond time scales. This information allows us to identify independent trends for each element, elucidating different stages within the phonon–phonon thermalization process. In combination with an element-specific breakdown of the phonon dispersion, we relate the experimental results to a phonon picture and identify a prolonged overpopulation of low-energy optical phonons.

A monolayer WSe_2_ sample was prepared by micromechanical exfoliation of a bulk crystal and subsequently transferred to a 10 nm thick amorphous Si_3_N_4_ membrane substrate by an all-dry transfer technique.^46^ The substrate is a 100 × 100 μm^2^ window, which is approximately the size of the probe beam, (see Figure 1a) within a silicon frame, such that the entire sample area is probed. Figure 1b presents a photoluminescence spectrum, exhibiting a pronounced A-exciton resonance at 1.66 eV, as expected.^47^Figure 1c presents a sketch of the 4 kHz room-temperature UED experiment.^48^ A photon pulse with *hv* = 1.65 eV (751 nm) optically excites the sample, followed by a 75 keV electron pulse arriving at a variable time delay, *t*. The electrons arrive normal to the sample surface, producing a transmission diffraction pattern on a detector, as in Figure 1d (a faint Scherrer ring is caused by the substrate). The excited carrier density generated by the incident 1.4 mJ cm^–2^ fluence is (4.3 ± 0.1) × 10^14^ carriers/cm^–2^ (see Supporting Information), which is much higher than the reported Mott density (∼10^13^ cm^–2^).^49,50^ This suggests that the majority of excited carriers behave as quasi-free carriers, so excitonic effects are not considered herein, despite the agreement between the photon energy and the exciton resonance.

**Figure 1:**
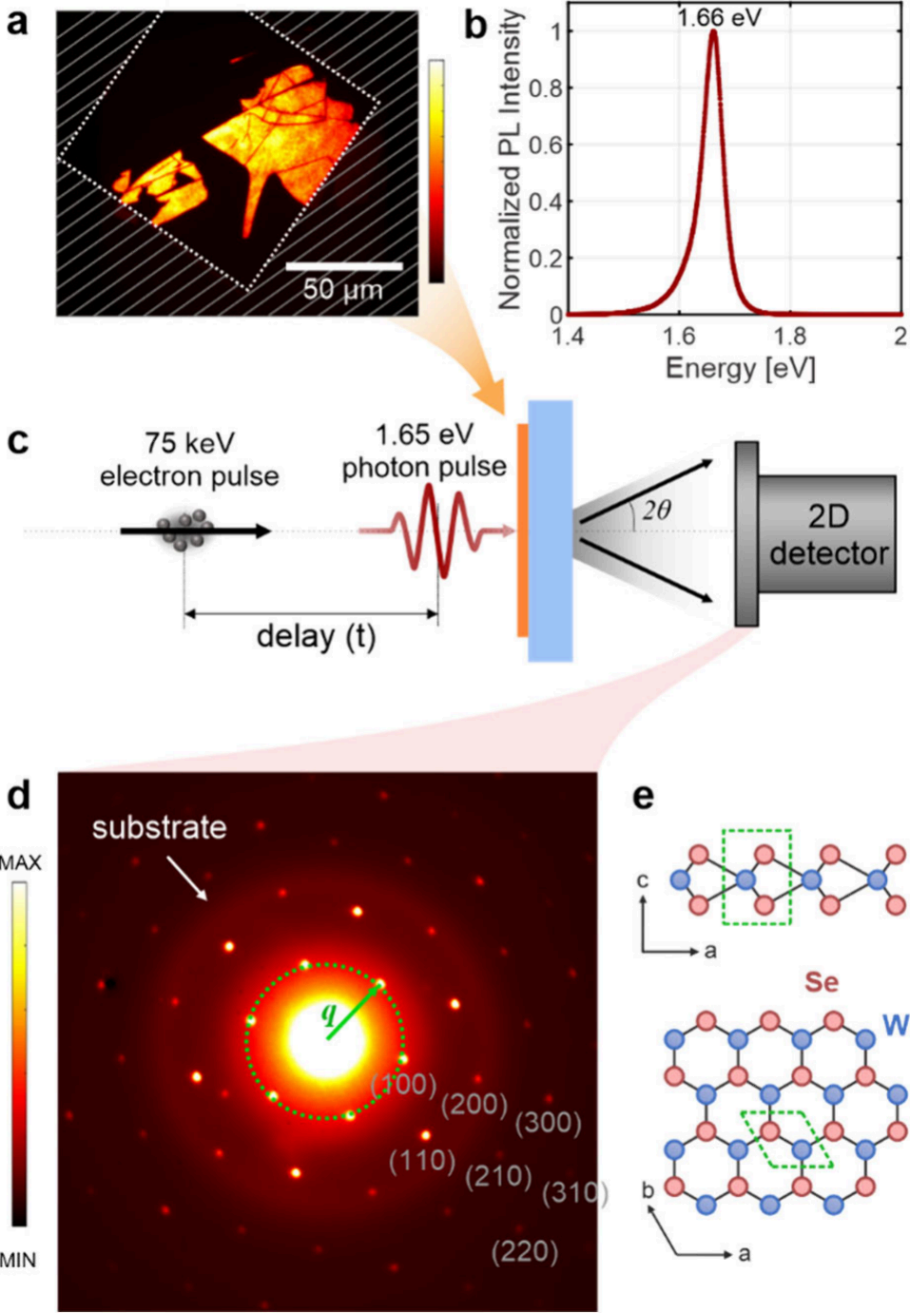
(a) A photoluminescence image of the sample on the 100 × 100 μm^2^ membrane window. The hatched area corresponds to thick silicon. (b) Photoluminescence spectrum of the sample. (c) Schematic experimental setup of ultrafast electron diffraction: an optical pulse excites the sample at nearly normal incidence. After a variable time delay an electron pulse transmits through the sample at normal incidence. The scattered electrons produce a diffraction pattern on a 2D detector at each time delay. (d) An example of such a diffraction pattern, exhibiting Bragg spots from the monolayer WSe_2_ and Scherrer rings from the substrate. One group of reflections with the same |***q***| is indicated. (e) Side and top-down views of the WSe_2_ monolayer structure, with the unit cell highlighted.

The observed Bragg reflections provide in-plane sensitivity and are denoted with Miller indices (*hk*0). Each Bragg reflection is described by a scattering vector ***q*** (Figure 1d). We define the scattering vector as |***q***| = 4*πλ*^–1^sin θ (λ is the electrons’ de Broglie wavelength and θ is the Bragg angle). For hexagonal monolayer WSe_2_, ***q*** satisfies
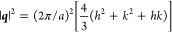
with *a* = 3.297 Å.^51^ Note that Bragg reflections with different Miller indices can have the same scattering vector length |***q***|.

We collect diffraction patterns for several pump–probe delays, and extract the intensities of the Bragg reflections, which are described analytically by^52,53^
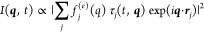
1

Here we sum over all three atoms in the monolayer unit cell (Figure 1e): *f*_*j*_^(*e*)^(*q*) are tabulated electron scattering factors of Se or W atoms,^54,55^*r*_*j*_ is the relative equilibrium position of the *j*th atom, and τ_*j*_ is its temperature factor (explained below). Since we do not observe a coherent response within our time resolution, we do not consider changes to *r*_*j*_, and subsequently eq 1 yields the same intensity for all (*hk*0) combinations that produce the same |***q***|. We therefore average the intensities of all reflections that share the same |***q***|. Figure 2a presents these averaged intensities.

**Figure 2:**
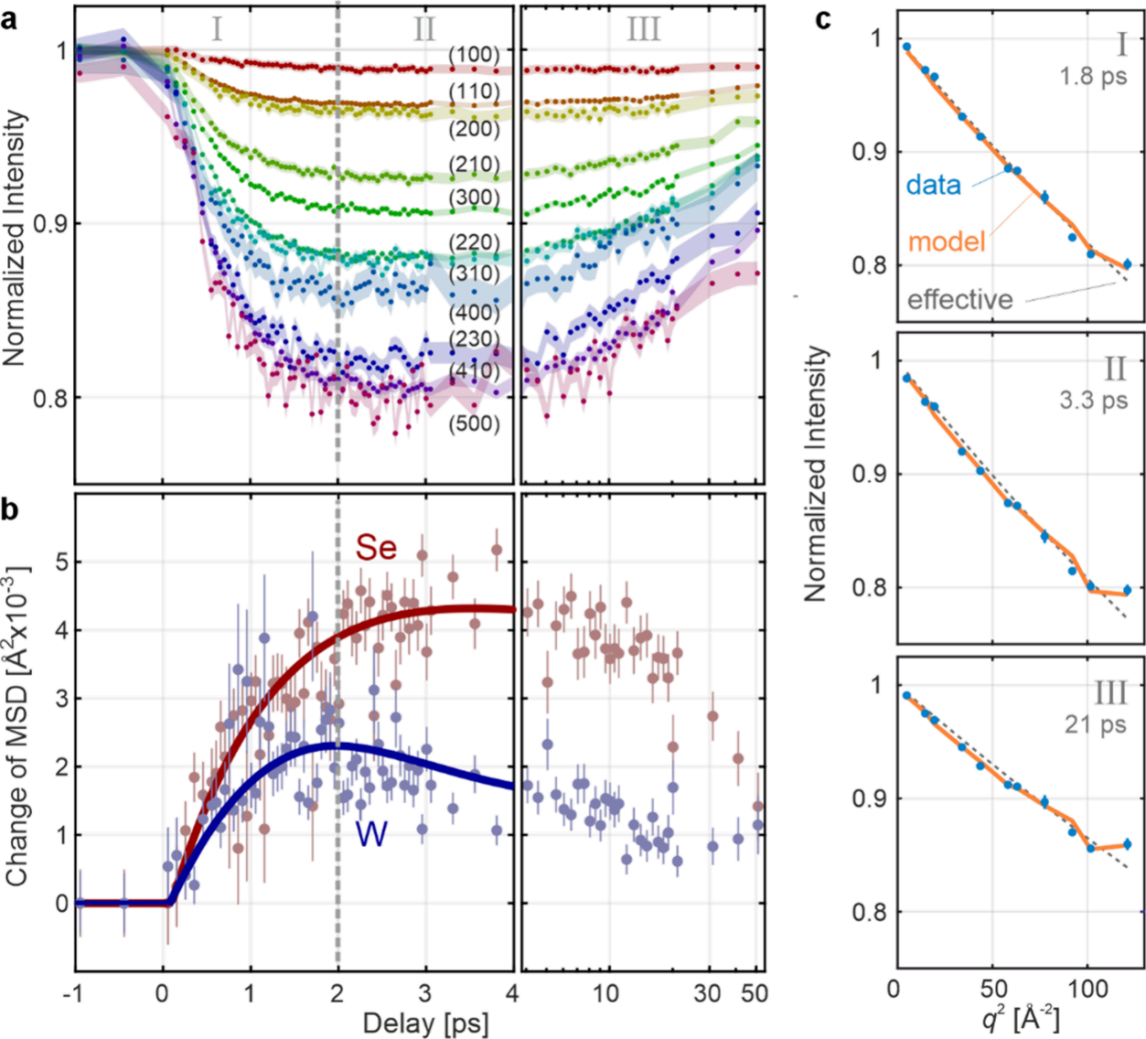
(a) Normalized Bragg reflection intensities as functions of pump–probe delay. Each curve represents the average of all reflections with the same scattering vector length and is denoted by a representative (*hk*0) combination. (b) The change in atomic mean-squared displacement (MSD) of each element (*ΔU*^(*W*)^ and *ΔU*^(*Se*)^), as extracted from all data in panel (a) using eq 1. The error bars are the fit standard deviations. The solid lines are drawn lines serving as guides to the eye. Time-ranges labeled I, II, and III indicate stages in the evolution of MSDs based on changes in their trends. (c) Normalized intensities as functions of *q*^2^, at three selected delays chosen to demonstrate the reliability of the element-resolved method. Data points are the same as in (a); unless seen, error bars are smaller than the data icons. The solid lines (labeled “model”) are calculated from the results of the two-level fit routine, shown in (b). The lines are in excellent agreement with the data, particularly at larger *q*^2^, where variations are most pronounced. Dashed lines represent “effective MSD” fits, which are not element resolved (see Supporting Information). Note that these agree with our model at early delays, when the both elements behave similarly.

Photoexcitation suppresses all intensities due to the growth of the incoherent phonon population. For a microscopic understanding, we use eq 1 to interpret Figure 2a, such that the temperature factors τ_*j*_ encode all dynamics. These factors express the Debye–Waller effect, through which Bragg reflections are attenuated with increased atomic vibrations (e.g., when heated). These vibrations are quantified using a time-averaged matrix **U**^(*j*)^, which describes the mean-squared displacement (MSD) of atom j around its equilibrium position, along different spatial directions (**U** was recently used in a UED study of black Phosphorus^29^). The MSD encodes all incoherent atomic vibrations, which are a superposition of all populated phonon modes.^52,53^ The temperature factor τ_*j*_ follows^53^
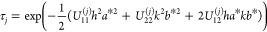
2

where a* and b* are the reciprocal lattice vector lengths. U_11_^(*j*)^, U_22_^(*j*)^, and U_12_^(*j*)^ are elements of **U**^(*j*)^ along different in-plane directions (this experiment is not directly sensitive to out-of-plane MSD contributions). Atoms’ in-plane local environment in monolayer WSe_2_ is the same as in bulk, so the same in-plane symmetry constraints on **U**^(*j*)^ apply (space group #194, with *l* = 0). For both the W and Se crystallographic sites, these dictate that *U*_11_^(*j*)^ = *U*_22_^(*j*)^ = 2*U*_12_^(*j*)^ ≡ *U*^(*j*)^.^52^ The in-plane MSD of each element is therefore described by a single parameter, *U*^(*j*)^, and eq 2 becomes
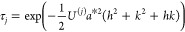
3

Note that use of τ_*j*_ is valid for describing nonthermal states, and its name “temperature factor” is purely nomenclature. The effect of photoexcitation is now expressed in the delay-dependent change of each element’s MSD, Δ*U*^(*j*)^(*t*). We therefore consider *U*^(*j*)^ as *U*^(*j*)^(*t*) = *U*_0_^(*j*)^ + *ΔU*^(*j*)^(*t*), in which *U*_0_^(*j*)^ is the unperturbed MSD. Finally, we insert *U*^(*j*)^(*t*) into τ_*j*_ (eq 3), and then insert τ_*j*_ into eq 1, producing an expression for *I*(**q**,*t*) which is unique for each ***q*** value. By plugging in all (*hk*0) combinations, we reach a set of unique equations for *I*(**q**,*t*) that all depend only on two dynamic variables: *ΔU*^(*W*)^(*t*) and *ΔU*^(*Se*)^(*t*), which are independent of ***q***. This means that each curve in Figure 2a can be described by the temporal evolution of these two quantities. Note that we assume only perturbative vibrational coupling to the substrate, such that the two Se atoms in the monolayer unit cell share the same Wyckoff position, and thus that *U*^(*Se*)^(*t*) is the same for both.

To extract the temporal evolution of both *U*^(*W*)^ and *U*^(*Se*)^ from the data, we employed a two-level routine that fits all observed intensities in Figure 2a to this equation set. First, for all delays, all *I*(**q**,*t*) must share the unperturbed MSD values *U*_0_^(*W*)^ and *U*_0_^(*Se*)^. Second, all *I*(**q**,*t*) at a *given* delay share the same pair of *ΔU*^(*W*)^ and *ΔU*^(*Se*)^ values. The optimal values obtained for *U*_0_^(*Se*)^and *U*_0_^(*W*)^ were 6.3 × 10^–3^ Å^2^ and 3.4 × 10^–3^ Å^2^, respectively, in agreement with experimental values reported for a similar material, TiSe_2_^56^ as well as with calculated values for monolayer WSe_2_.^30^

Figure 2b presents the resulting evolution of *ΔU*^(*W*)^(*t*) and *ΔU*^(*Se*)^(*t*). To assess the quality of this method, Figure 2c presents intensities at selected delays as functions of *q*^2^. At first glance the curves appear linear, but deviations from linearity are clearly seen. The solid line is calculated from the results in Figure 2b. It successfully captures these deviations, as well as the delay-dependent differences between them, which are key to our method. The deviations appear more pronounced at higher *q*^2^, where relative errors are higher (Figure, 2a). This is reflected in the rather large error bars in Figure 2b, and at a few delay points for which poor convergence is apparent (e.g., near 1 ps in Figure 2b). Nevertheless, the overall trends and differences between the two elements in Figure 2b are clear and attempts to fix other ratios between W and Se MSDs cannot reproduce the data (see Supporting Information). We conclude that the data in Figure 2b reliably represents an element-specific perspective of the evolution of incoherent atomic vibrations.

The solid lines in Figure 2b are presented to highlight trends in the *ΔU*^(*W*)^ and *ΔU*^(*Se*)^ data. To avoid suggestive interpretation, we note that the same trends emerge when the data are smoothed with a simple least-squares approach (see Supporting Information). By inspecting the changes in trends exhibited by *ΔU*^(*W*)^ and *ΔU*^(*Se*)^ (Figure 2b), we observe three distinct stages:

I. *t* < 2 ps: both W and Se exhibit a rapid increase in MSD.
II. 2 < *t* < 4 ps: opposite trends emerge, as the MSD of Se keeps growing, while that of W decreases rapidly.
III. *t* > 4 ps: all MSD evolution processes slow down, as the MSD of Se decreases, while the rapid decrease of W’s MSD slows down and is eventually overtaken by slight growth at ∼20 ps (see Supporting Information for dynamics on a linear time delay scale)

Variations in MSD can be described as an evolution of the phonon population (see Supporting Information). According to ref (17), when semiconductors are photoexcited and undergo electron cooling, intravalley carrier scattering with smaller momentum and higher energy tends to occur, favoring stronger coupling to optical phonons near the Γ point, than to other phonon groups. This is supported by calculations on bulk WSe_2_^36^ and is also consistent with reports on other monolayers, e.g. graphene and MoS_2_.^18,57^ It is then likely that phonon thermalization in monolayer WSe_2_ starts with overpopulating higher energy optical modes near Γ, followed by populating lower energy modes instead via phonon–phonon scattering. The continuous increase in *ΔU*^(*Se*)^ compared to the decrease of *ΔU*^(*W*)^ in stage II suggests a preferential growth in the population of phonon modes dominated by Se vibrations, and cannot agree with an increase in lattice temperature. As such, stage II is direct evidence that the lattice is in a nonthermal state.

For further interpretation in terms of phonons, we conducted a density functional perturbation theory (DFPT) calculation using the QUANTUM ESPRESSO package.^58^ A fully relaxed atomic structure was adapted using the plane-wave self-consistent field program. The electronic ground state is evaluated using a 10 × 10 × 1 mesh (convergence threshold: 1 × 10^–9^ Ry/Bohr). Subsequently, the phononic structure is calculated for the dynamical matrices on an 8 × 8 × 1 *q*-grid (accurate consistency threshold: 1 × 10^–14^ Ry/Bohr).

Figure 3a presents the calculated phonon dispersion of monolayer WSe_2_, and is in agreement with previous reports.^59,60^ Out-of-plane polarized phonon modes are depicted in gray. Our experiment is nearly insensitive to them because they have very small in-plane contributions. Figure 3b presents the corresponding phonon density of states (DOS; dotted line). The DOS was subdivided twice: polarization dependence (in- and out-of-plane) and elemental dependence (W or Se vibrations). Figure 3b presents in-plane DOS components: total in-plane (solid line), and element-specific (shadings). We find that in-plane polarized states account for most of the DOS (except at the highest energies). These are vibrations to which our experiment is directly sensitive (eq 2).

**Figure 3:**
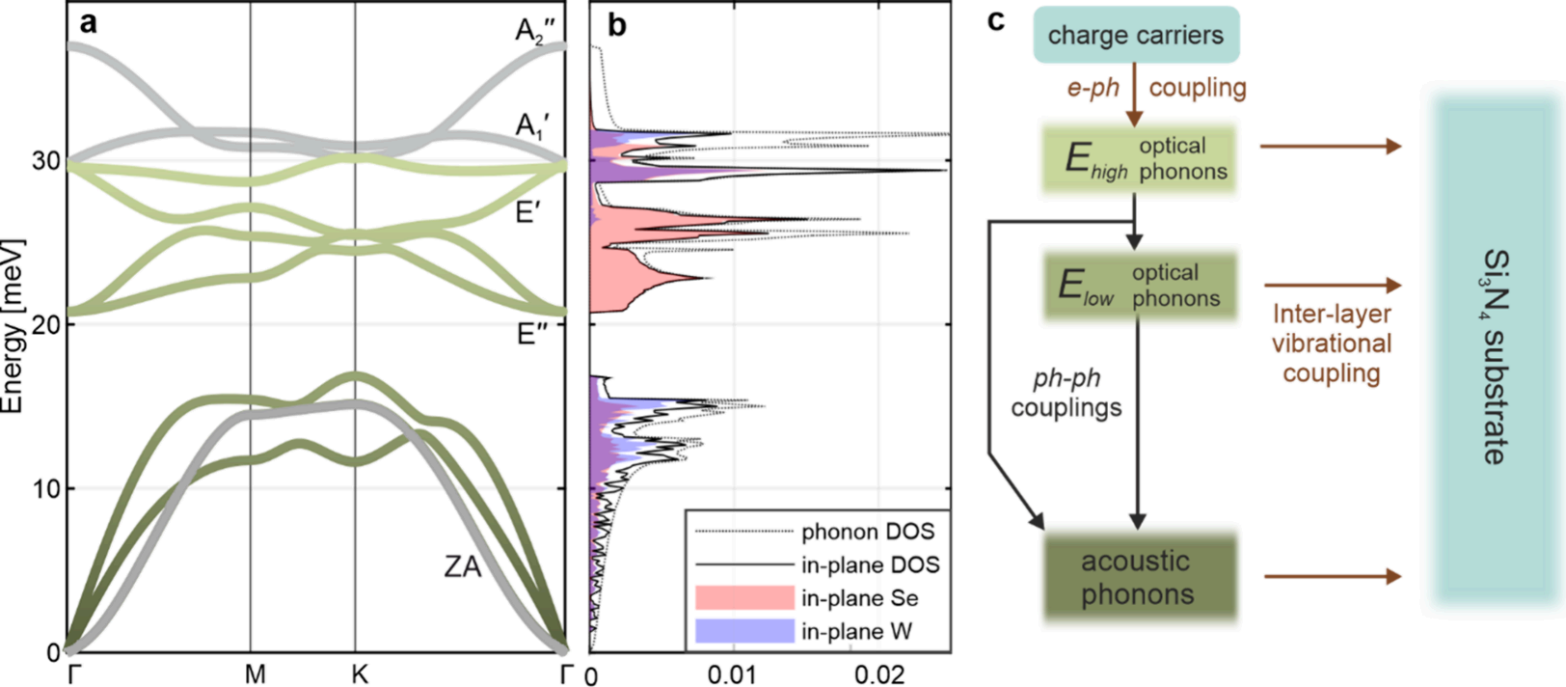
(a) Calculated phonon dispersion of monolayer WSe_2_. Gray-colored bands are polarized out-of-plane, so the experiment is nearly insensitive to them (eq 2). (b) Phonon density of states (DOS): total (dotted line), total in-plane (solid line), and breakdown of in-plane DOS by atom type (shades of red and blue). (c) Energy flow diagram illustrating phonon thermalization. The color scheme relates to the bands in (a).

The key result of this calculation is the element-specific breakdown of in-plane vibrations, as it relates to element-resolved experimental results in Figure 2b. By combining the two, we can interpret which phonons are dominant in each stage of thermalization. The primary observation from Figure 3b is that in-plane Se vibrations dominate the lower-energy range of optical phonons (*E*_*low*_ ≈ 21 to 27 meV), while W vibrations are notably absent. In other energy ranges, including the acoustic branches, W vibrations contribute similarly to Se. This is consistent with calculations on other monolayer TMDCs.^61^

Based on Figure 3b, we now further interpret the stages in the MSD evolution (Figure 2b). To guide this, we present an energy flow diagram (Figure 3c). In stage I, the similar increases in *ΔU*^(*Se*)^ and *ΔU*^(*W*)^ suggest an initial growth in occupation of the high-energy optical branches (*E*_*high*_ > ∼27 meV), particularly in states near Γ (low momentum), where Se and W vibrations contribute similarly (Figure 3b). This occurs due to energy flow from the excited carriers via EPC. A relatively small growth in zone-boundary acoustic phonons is also expected.^36^ In stage II, the dominance of Se vibrations over W (Figure 2b) suggests a growth in the *E*_*low*_ optical phonons (dominated by Se, Figure 3b) through a loss of *E*_*high*_ phonons via phonon–phonon scattering. The data suggest that a preferentially high population of these Se-dominated phonons persists for several ps. Lastly, in stage III an unambiguous observation here is that the intensity of all Bragg reflections recovers (Figure 2a), indicating a weakening of the Debye–Waller effect. This is typically interpreted as due to energy flow away from the excited sample volume as part of a slow thermalization of the whole system back to the original equilibrium. Indeed, in stage III the W vibrations approach those of Se, which drop faster (Figure 2b; see Supporting Information). This can indicate a growth in the acoustic phonon population because both elements contribute similarly to them (Figure 3b), unlike the *E*_*low*_ phonons.

Critically, our data suggests another energy flow process. This is because the highest MSD values are observed at the transition from stage II to stage III. If energy is indeed flowing from the *E*_*low*_ phonons into acoustic phonons, we expect a further increase in MSD, because MSD typically scales as *E*^–1^,^52,53^ implying that acoustic phonons contribute most to MSD amplitudes. Furthermore, energy conservation would dictate that a high-energy phonon creates multiple low-energy phonons, each contributing more to MSD. However, as the Se-dominated signature decreases, so does the overall MSD, which is inconsistent with this expectation. We consider two possibilities.

The first is energy flow through vibrational coupling to the substrate, which is not photoexcited because of its large band gap. Furthermore, the Si_3_N_4_ phonon dispersion greatly exceeds the energy range in Figure 3a, and within this range, its bands are dense with no energy gaps.^62^ This suggests that coupling can occur from any WSe_2_ optical phonons, diverting energy away from the sample before it reaches the acoustic phonons, effectively “shunting” their expected population growth (Figure 3c). Energy deposited in acoustic phonons via EPC is likely also diverted in this way. The recovery of all Bragg reflections (Figure 2a, stage III) supports this explanation, as does an observed attenuation of the substrate’s Scherrer rings (stage II; see Supporting Information), indicating that such vibrational coupling does indeed occur. This implies that “acoustic phonon shunting” may serve as a novel approach toward heat management in future nanoscale technology, with one layer conducting charge and another conducting heat.

Another possibility is generation of out-of-plane polarized phonons, to which our experiment is largely insensitive. These include ZA phonons, which can disperse at lower energies than all other modes.^18,63^ The recovery at the later delays in Figure 2a would then reflect a weakening of in-plane vibrations, but not of the total vibrations, as the system continues to thermalize. We note that ultrafast occupation of out-of-plane modes in monolayers is debated.^18,30,63^

In summary, we used UED to probe photoinduced lattice dynamics in monolayer WSe_2_. Each element’s amplitude of incoherent vibrations was quantitatively extracted as a function of delay, producing an element-specific view of the vibrational response to photoexcitation. From differences between the atom species’ vibrational responses, we identify stages in phonon–phonon thermalization. By combining this with an elemental breakdown of the phonon dispersion, we present a scenario in which an initial high-energy phonon population is generated, followed by the preferential population of low-energy optical phonons, which persists for several ps. This is followed by a rapid recovery of all Bragg intensities, which disagrees with the arrival at an elevated thermal phonon distribution, because an increase of acoustic phonons is absent. We discuss two explanations: energy flow from the optical phonons directly to the substrate, or to ZA phonons, to which we are insensitive. The former explanation may serve as a route for heat management in nanoscale devices by shunting acoustic phonon generation, and may be elucidated by future experiments on free-standing monolayers. Beyond the specific case of WSe_2_, the approach to time-resolved diffraction demonstrated here provides an element-resolved view of ultrafast phonon–phonon interactions. This can be readily applied to other materials, particularly when certain atoms are associated with specific effects like magnetism. With further computation, phonon-branch-specificity is also conceivable. This real-space view is complementary to the momentum-resolved view of diffuse scattering. Both serve to elucidate vibrational energy flow, which can ultimately lead to more efficient thermal management.

## Supporting Information Available

Analysis and methods used in this study (PDF)
