# Supplementary material for: Element-Specific Ultrafast Lattice Dynamics in Monolayer WSe2
Source: Nano Lett. 2024 Oct 21;24(43):13671–7. doi: 10.1021/acs.nanolett.4c03611 (PMC11528438; doi:10.1021/acs.nanolett.4c03611)
Supplement: Supplementary file 1 — nl4c03611_si_001.pdf [file nl4c03611_si_001.pdf]

Element-specific ultrafast lattice dynamics in monolayer WSe_2

Supplementary Information

Hyein Jung[1,2], *Shuo Dong*[2,a], *Daniela Zahn*[2,b], *Thomas Vasileiadis*[2,c], *Helene Seiler*[2,d], *Robert Schneider*[3], *Steffen Michaelis de Vasconcellos*[3], *Victoria C. A. Taylor*[2], *Rudolf Bratschitsch*[3], *Ralph Ernstorfer*[1,2], *Yoav William Windsor*[1,2]

[1] Institute for Optics and Atomic Physics, Technical University Berlin, Strasse des 17, Juni 135, 10623 Berlin, Germany

[2] Department of Physical Chemistry, Fritz Haber Institute of the Max Planck Society, Faradayweg 4-6, 14195 Berlin, Germany

[3] Institute of Physics and Center for Nanotechnology, University of Münster, Heisenbergstraße 11, 48149 Münster, Germany

Contents

1 Excited carrier density ... 2

1.1 Absorbed fluence ... 2

1.2 Calculation of the excited carrier density ... 2

1.3 Transfer matrix method ... 2

1.4 Transmission measurement as a test of the transfer matrix method's reliability ... 3

2 Full linear scaled element-specific dynamics ... 6

3 Lattice dynamics of the substrate (Si_3N_4 membrane) ... 7

4 Relating mean squared displacements with phonons ... 9

5 Fit routine for extracting time-dependent MSDs ... 10

5.1 Introduction ... 10

5.2 Signatures of the element-specific MSDs ... 12

5.3 Element-specific MSD fit procedure ... 12

5.4 The limit of sensitivity to $\boldsymbol{U}_0$ ... 13

5.5 Comparison with unperturbed MSD calculated in the study of I.-C. Tung et al. ... 14

6 Comparison to previously reported monolayer WSe_2 data ... 16

7 References ... 17

1 Excited carrier density

In this section we explain the calculation of the excited carrier density. We also perform a simple light transmission measurement as a test for the reliability of our method in the case of a monolayer sample.

1.1 Absorbed fluence

The following calculations are necessary to deduce the photoinduced carrier density. When the pump pulse excites the sample, part of the beam is reflected, part is transmitted, and part is absorbed. Knowledge of the fluence that is actually absorbed in the sample is required to approximate the carrier density. An incident power of P_{in} = 4.3 ± 0.1 mW was measured during the measurement with a repetition rate of f = 4 kHz. The pump profile, depicted in Figure S1a, was imaged and approximated to a 2D gaussian $G(x,y)$, with a full-width-half-maximum of 250 µm and an integrated power of 4.3 mW. Next, a binary image of sample area from Figure 1a in the main text was generated (inset in Figure S1a). The integrated sample area was calculated as $A = 0.281 \times 10^{-4}$ cm^2. We assume an optimal spatial pump-probe overlap, such that the center of the 2D gaussian was aligned with the center of mass of the sample's binary image (denoted $S(x,y)$), as shown in Figure S1a. Consequently, the effective pump power P_{eff} that impinges on the sample is estimated as (see also Figure S1b):

$$p_{eff} = \int dxdy\; \delta\big(S(x,y)\big)\, G(x,y) \;= 0.165 \pm 0.004\, mW$$

The incident fluence is then estimated as $I_{inc} = \frac{P_{eff}}{A \cdot f} = 1.466 \pm 0.034\; mJ/cm^2$, impinging at nearly normal incidence on the sample. The absorbed fluence I_{ab} is defined as:

$$I_{ab} = (1 - R - T) \times I_{inc} = 0.114 \pm 0.003\; mJ/cm^2,$$

with R (= 0.0059) and T (= 0.9161) being the reflectance and the transmittance respectively. (The absorption is then ~ 0.08) These values were obtained using the transfer matrix method (explained below) and are comparable to the experimental literature value for absorption at room temperature with a similar incident angle [1,2]. The optical constants used for the transfer matrix were extracted from ref. [3] using an online tool [4].

1.2 Calculation of the excited carrier density

The excited carrier density in a single layer can be estimated as $n = I_{ab}/E_{ph}$ [5], with E_{ph} the photon energy of the pump beam. In our case we use 1.65 eV (= 2.64×10^{-16} mJ) photons, giving n = (4.3 ± 0.1) × 10^{14} carriers/cm^2, assuming one carrier excitation per absorbed photon.

1.3 Transfer matrix method

In nanoscale samples, interfacial effects become significant, and should be considered in the reflection and transmission calculation. In thin films, the incident wave experiences multiple reflection and transmission events at the interfaces. These partially transmitted (or reflected) beams can interfere with each other, and the total transmission (or reflection) of the film can be obtained by integrating an infinite number of transmissions and reflections, considering their interference.

Therefore, we implemented a method which describes the film using a simple matrix operation based on the continuity conditions in Maxwell's equations. [6] This method calculates the total transmission and reflection using a matrix called the "transfer matrix". The transfer matrix M is given by:

$$M = \begin{pmatrix} cos(nkd) & \frac{sin(nkd)}{nk} \\ -nk\,sin(nkd) & cos(nkd) \end{pmatrix},$$

where n is the (complex) index of refraction (extracted from ref. [3] using an online tool [4]), k is the wave number of the incident beam inside the material, and d is the thickness of the sample, in our case we took half the c axis length, taken from ref. [7]. For simplicity, we neglect the substrate (Si_3Ni_4 membrane), and assume vacuum on both sides of the sample. We expect interference effects from the substrate to be negligible as it is transparent our pump beam due to its large band gap (~ 4.5 eV [8]).

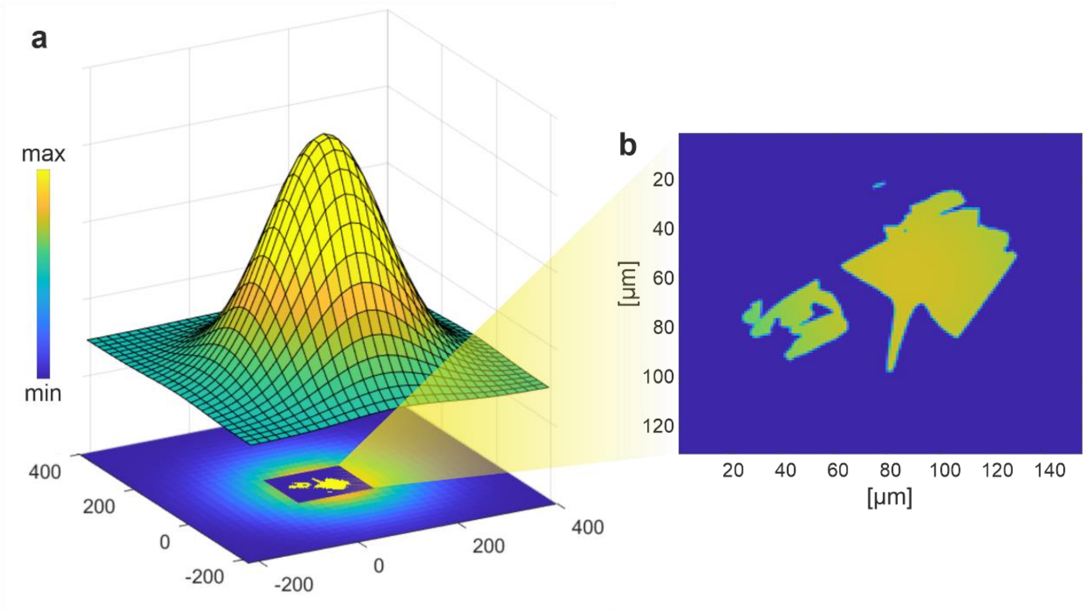

Figure S1 (a) The pump beam's profile represented as a 2D gaussian, with its shape projected onto the sample's binary image, which is at the center of the peak. The axes are in units of µm. (b) Pump intensity profile overlayed onto the sample area. The effective pump power is estimated by integrating this image.

1.4 Transmission measurement as a test of the transfer matrix method's reliability

To confirm the reliability of employing the transfer matrix method on monolayer WSe_2, we used it to estimate the thickness of the sample and compared that to a literature value (half the c axis lattice constant). For this, we used a monochromatic camera connected to a microscope with two wavelength filters: 410 nm and 650 nm, both having a ~20 nm bandwidth. The filtered light was transmitted through the sample and into the microscope objective. The camera's gamma correction was set to 1, such that the brightness of the object is linear to the pixel values. Additionally, no region on the camera was saturated. The transmission measurements were carried out as follows:

1. Images of the sample were taken using each filter, as presented in Figure S2(a,b). Here, the brightest area is the bare Si_3N_4 membrane (transparent at these wavelengths) and the dark background is thick silicon, which does not transmit at all.
2. A histogram of transmitted intensity is presented as an inset to each image. Three Gaussians are observed: (a) the darkest one is from the thick Si, from which the camera's dark background is extracted, (b) a brighter one from the sample itself, and (c) the brightest one from bare areas of the transparent substrate, from which the incident intensity is deduced. Gaussian fits were used to find the peaks' maxima (solid lines in the insets).
3. The image's intensities are converted into relative transmitted intensity: the peak center of the dark Gaussian (thick Si) is subtracted from every pixel's intensity, which are then divided by the center of brightest gaussian (from which the dark peak was subtracted). As such, the transmitted signal at the substrate is 1 and at the thick Si it is 0.

The transmission rates at the sample from each filter are summarized in Table 1.

Table 1 - experimental transmittance values and the corresponding thicknesses from the transfer matrix calculation

Filter	Transmission rate	Estimated thickness [nm]
410nm	0.796 ± 0.002	~0.68 ± 0.01
650nm	0.959 ± 0.002	~0.64 ± 0.03

We now use these values to test the calculation. Using the optical indices from ref. [3,4], the transfer matrix method was used to calculate transmission as function of sample thickness from each filter. This is plotted in Figure S2(c). The experimental transmission values are indicated for each filter, and their estimated sample thicknesses are marked at the intersection with the corresponding curve, and are listed in Table 1. They are in good agreement with each other, as well as with the $c/2 \approx 0.65\ nm$ value of the multilayer WSe_2 unit cell [7]. The final (averaged) thickness estimate is 0.66 ± 0.02 nm.

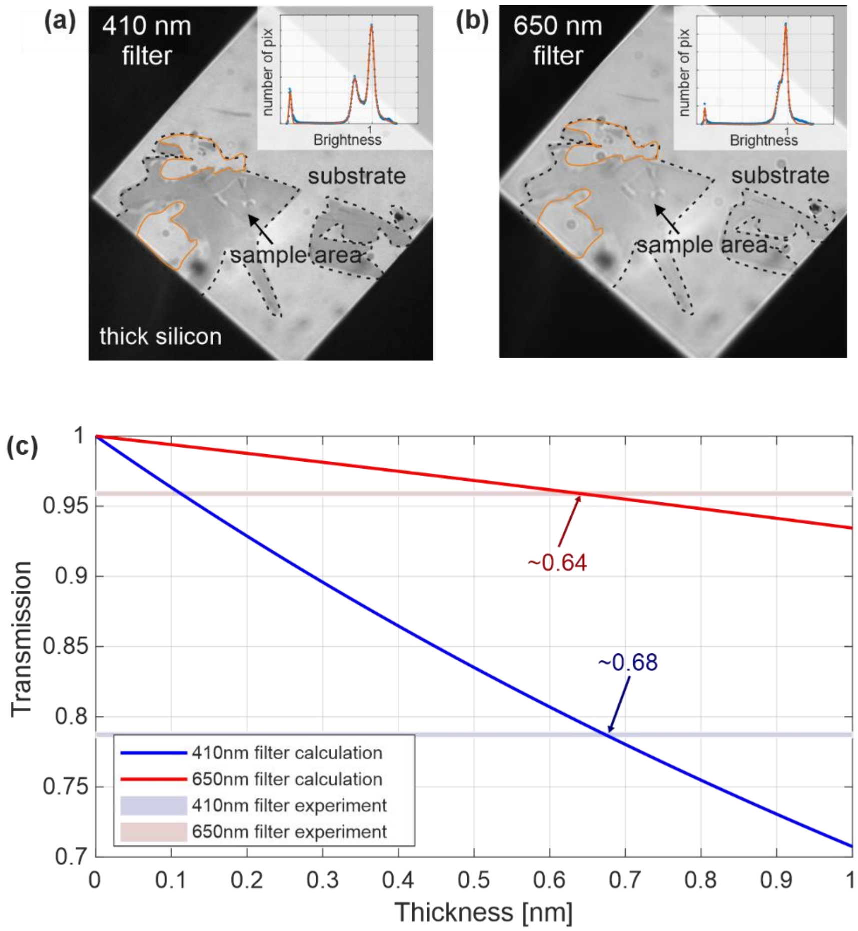

Figure S2 (a, b) grayscale images taken through a microscope in transmission mode, each using a different wavelength filter. The sample area is marked by dashed lines. The insets present a histogram of each image as blue dots, and the red curve is a 3-gaussian fit. Note that there is a slight discrepancy with the photoluminescence image in Figure 1a of the main text (orange marked area): these images were taken long after the experiment, and some areas appear to have degraded, (orange highlighted areas). (c) calculated transmittance of monolayer WSe_2 from each filter (solid curves) as function of sample thickness. Horizontal shaded lines represent the experimental transmittance values. Their thickness represents their certainty.

2 Full linear scaled element-specific dynamics

Figure S3a presents the same data as in Figure 2b of the main text on a linear horizontal scale. This highlights the trends of stage III, as mentioned in the main text, particularly in the W data at the latest delays. Additionally, Figure S3b presents the same data as Figure 2b and on the same horizontal scale, but the solid guidelines shown are smoothed interpolated curves from the $\Delta U^{(Se)}$ and $\Delta U^{(W)}$ data. This is presented as an additional measure of confidence in the trends we identify in stage I and II in Figure 2b, as it highlights the deviations of the data from the smooth guidelines (thick lines).

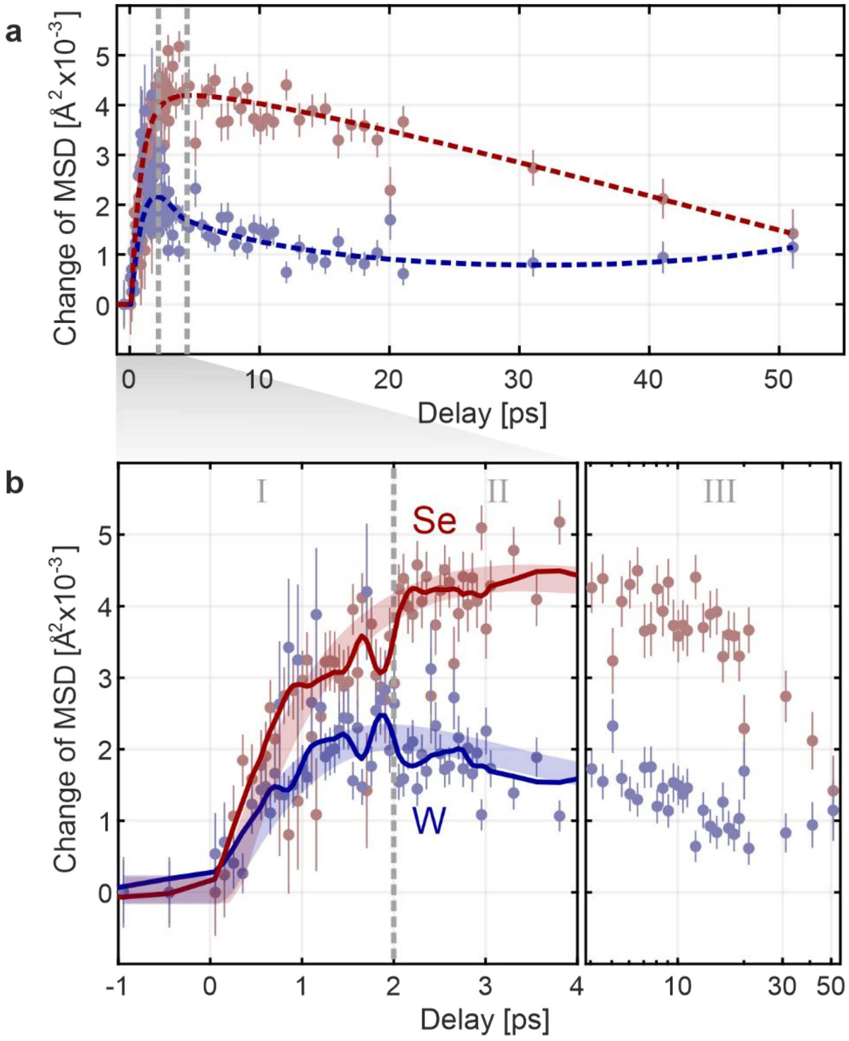

Figure S3 (a) The same data as in Figure 2b in the main text, but on one linear scale. The dashed lines are guides to the eye. (b) The same as Figure 2b in the main text, but instead of the solid guidelines, here the solid lines are a smoothed interpolation of the data. These are presented to highlight the validity of the trends we identify in the data, despite the existence of isolated delay points that sharply deviate.

3 Lattice dynamics of the substrate (Si_3N_4 membrane)

Here we analyze the faint ring pattern in our data, which originates from the polycrystalline substrate. The purpose of this is to investigate the lattice response of the substrate, most likely induced by energy flow from the sample, since the substrate does not absorb the pump pulse directly.

An example of an unpumped pattern is presented in Figure S4a, with the area excluded from the following analysis indicated. All Bragg reflections from the WSe_2 are also masked. For each delay, we generate a radial average of intensity around the direct beam, resulting in a radial distribution of intensity, as in Figure S4b. A peak shape is clearly observed, on top of significant radially decaying background.

We approximate this background as a third order polynomial function as shown in Figure S4b, and subtract it from the data at each delay. The area of the peak shape was then extracted using the best fit to a Gaussian (Figure S4c), producing a relative intensity $I_{rel}^{Si_3N_4}$. A delay dependent *effective* MSD of the substrate is then calculated as:

$$\Delta\langle U_{Si3N_4}^2\rangle = -6 \times \frac{log\left(I_{rel}^{Si_3N_4}\right)}{|q|^2}.$$

$\langle U_{Si3N_4}^2\rangle$, presented in Figure S4d, exhibits an initial rise until ~ 4 ps, after which it stabilizes to a near-constant value within the measured delay window of up to 50 ps. This can be attributed to an increased population of phonons in the substrate via interlayer vibrational coupling. Also presented is its best fit to an exponential rise of the form $A\,\exp(-\,\Delta t/\tau)$, multiplied by a Heaviside function. This produces a time scale of $\tau = 1.28 \pm 0.63$ ps.

Lastly, we comment on the possible effect of interfacial Coupling between the lower Se ions and the substrate. The interfacial coupling between the lower Se ions and the substrate could contribute to the observed dynamics. However, the interaction between the substrate and WSe_2 is through van der Waals forces. These forces are weak compared to covalent bonding within the WSe_2 layers and are unlikely meaningfully affect the phonon eigenmodes of the WSe_2 crystal. Therefore, we assume that the symmetry of the WSe_2 crystal is preserved despite the presence of the substrate, such that the Se ions on both side of the monolayer sit at crystalographically equivalent sites

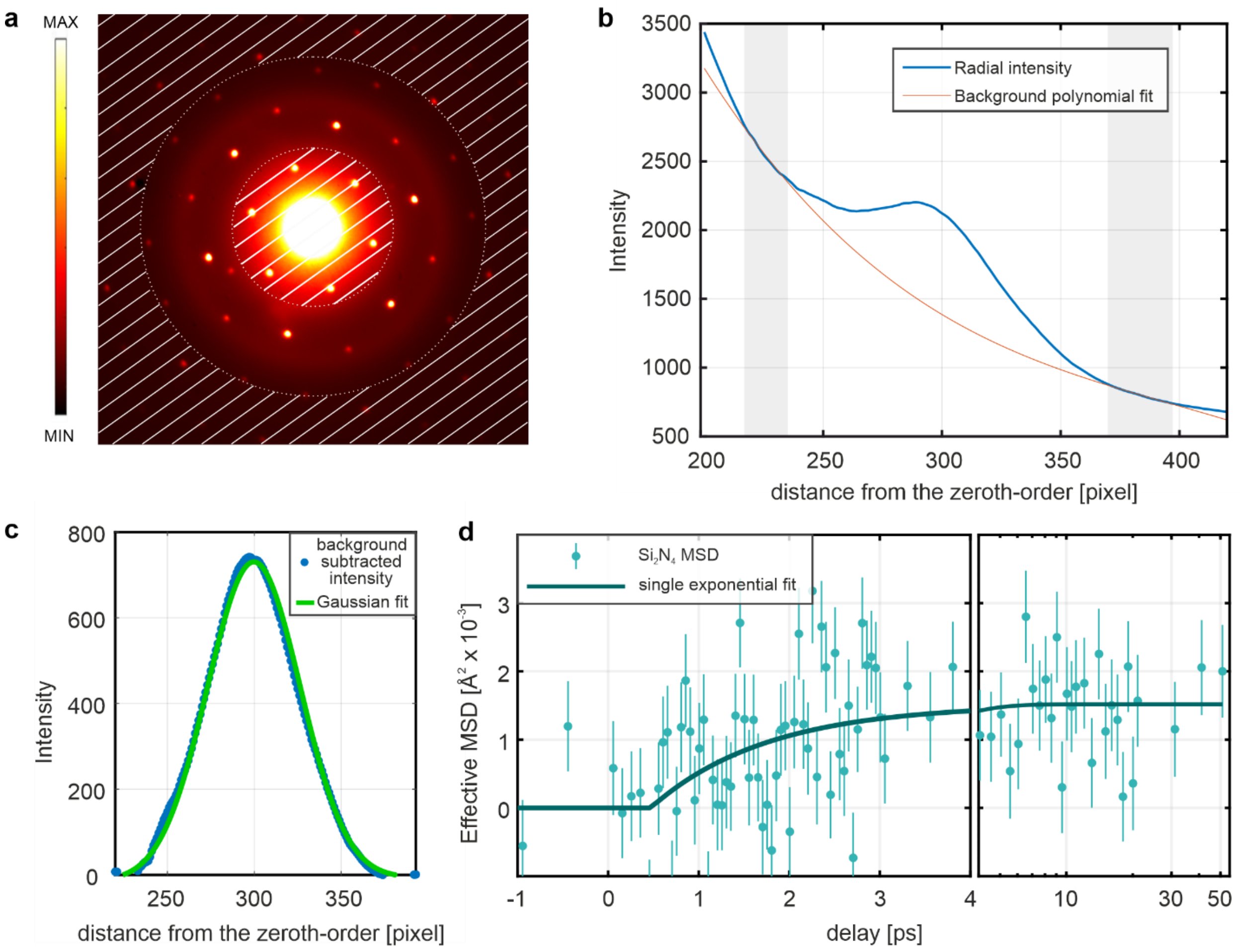

Figure S4 (a) An unpumped Bragg pattern, with the regions excluded from the azimuthal average indicated. (b) The radial distribution of intensity, extracted from the non-thatched area in (a). A background polynomial fit is also shown, estimated using the intensity in the gray-shaded. (c) Example of background-subtracted radial intensity from (b) with a Gaussian fit. (d) The delay dependence of the change in effective MSD of the substrate, extracted from fits as in (c), at each delay. The error bars are the fit standard deviations. The solid curve represents a single exponential fit convoluted with a Heaviside function.

4 Relating mean squared displacements with phonons

In this section we relate the measured mean squared atomic displacements (MSD) with the phonons in the system. The atomic oscillations (treated within the limit of a harmonic approximation) can be understood as a combined consequence of all populated phonon modes.

Atomic vibrations cause the precise position of each atom in the periodic lattice to smear out, reducing the intensity of Bragg reflections. This is the Debye-Waller effect, which can be described by the "temperature factor" term in Eq. 1 of the main text: [9,10]

$$\tau_j = \exp\left(-\frac{1}{2}\langle(\boldsymbol{q}\cdot\boldsymbol{u}_j)^2\rangle\right)$$

Here $\boldsymbol{u}_j$ is the instantaneous displacement of the atom j from its equilibrium position due to vibrations. In a harmonic approximation, the vibrational modes are independent of each other. Therefore, the instantaneous distance $\boldsymbol{u}_j$ can be described as the sum of contributions of all populated phonon modes: [11]

$$\boldsymbol{u}_j = \mathrm{Re}\left[\frac{1}{\sqrt{\mu_j}}\sum_{\beta,\boldsymbol{k}}\left[a_{\beta,\boldsymbol{k}}\boldsymbol{e}_{\beta,\boldsymbol{k}}\exp\left(i\boldsymbol{k}\cdot\boldsymbol{r}_j - i\omega_{\beta,\boldsymbol{k}}t - i\delta_{\beta,\boldsymbol{k}}\right)\right]\right],$$

where μ_j is the mass of the atom j, $a_{\beta,\boldsymbol{k}}$ is the amplitude of the βth phonon branch with the phonon momentum $\boldsymbol{k}$, $\boldsymbol{e}_{\beta,\boldsymbol{k}}$ is the phonon polarization vector, $\omega_{\beta,\boldsymbol{k}}$ is the phonon frequency, and $\delta_{\beta,\boldsymbol{k}}$ is an arbitrary phase factor indicating incoherent phonons.

Here, $a_{\beta,\boldsymbol{k}}$ is directly related to the MSD and can be written as a function of occupation number of a phonon mode $n_{\beta,\boldsymbol{k}}$. This will now be shown in a series of steps: first, we calculate the total mean kinetic energy of the vibrating lattice and relate it to the total mean energy of the phonon system: [10,11]

$$\bar{E}_{kin} = \frac{1}{2}\sum_j \mu_j\langle\dot{\boldsymbol{u}}_j^2\rangle = \frac{1}{4}\sum_{\beta,\boldsymbol{k}}\left|a_{\beta,\boldsymbol{k}}\right|^2\omega_{\beta,\boldsymbol{k}}^2.$$

Based on the quantum theory of harmonic oscillators, the total mean energy of the phonon system is given by:

$$\bar{E} = 2\bar{E}_{kin} = \sum_{\beta,\boldsymbol{k}}\hbar\omega_{\beta,\boldsymbol{k}}\left(n_{\beta,\boldsymbol{k}} + \frac{1}{2}\right).$$

By comparing the last two equations, the amplitude of the total populated phonon modes can be written as: [10]

$$\left|a_{\beta,\boldsymbol{k}}\right|^2 = \frac{2\hbar}{\omega_{\beta,\boldsymbol{k}}}\left(n_{\beta,\boldsymbol{k}} + \frac{1}{2}\right).$$

Finally, the atomic mean-squared displacement (MSD) is the sum of all amplitudes $a_{\beta,\boldsymbol{k}}$ of the occupied phonon modes, allowing it to be expressed as: [10]

$$\langle \boldsymbol{u}_j^2 \rangle = \frac{1}{2\mu_j} \sum_{\beta,\boldsymbol{k}} |a_{\beta,\boldsymbol{k}}|^2 = \frac{1}{\mu_j} \sum_{\beta,\boldsymbol{k}} \frac{\hbar}{\omega_{\beta,\boldsymbol{k}}} \left(n_{\beta,\boldsymbol{k}} + \frac{1}{2} \right).$$

It is important to note from this last step that the change of MSD is sensitive to the change of phonon distribution, as the sum in the expression for $\langle \boldsymbol{u}_j^2 \rangle$ includes all populated phonon modes $n_{\beta,\boldsymbol{k}}$. Additionally, in the main text, we discuss about the MSD in terms of $\mathrm{U}_{nm}^{(j)}$ (instead of $\langle \boldsymbol{u}_j^2 \rangle$), which is the element of the U-matrix. The U-matix terms and the MSD relate according to $\langle \boldsymbol{u}_j^2 \rangle_{nm} = \hat{\boldsymbol{e}}_n{}^T \boldsymbol{U}^{(j)} \hat{\boldsymbol{e}}_m = U_{nm}^{(j)}$, where the n and m correspond to the crystal axes 1, 2, or 3. [9,10] Therefore, the element-specific MSD at each time delay in the main text ($\mathrm{U}_{nm}^{(j)}(t)$, with $j =$ W or Se) represents a weighed sum of all every phonon's displacement amplitude on atom *j* at a given time delay.

In conclusion, through the temperature factor τ_j, Bragg reflections provide sensitivity to the effect of phonons throughout the entire Brillouin zone. [9]

5 Fit routine for extracting time-dependent MSDs

This section details the procedure used for extracting delay-dependent MSDs for each element.

5.1 Introduction

The intensity of a Bragg reflection is analytically described by Eq. 1 of the main text. Each measured Bragg reflection is characterized by unique Miller indices ($h\ k\ l$). By plugging these into Eq. 1, we find that the intensity of each family of Bragg reflections is described as a unique equation (in this work we probed 10 unique families).

By Plugging in Eq. 3 into Eq 1, we find that these equations depend on $U^{(j)}$, the MSD of the jth ion in the unit cell. Since there are only 3 ions in the monolayer unit cell (two Se, one W), and the two Se ions sit at symmetry-equivalent sites, the index j can only have two values, which we name W or Se. Since phonons are the only considered source of a pump-induced intensity changes, we now see that all 10 unique equations depend on only two time dependent terms: $U^{(\mathrm{W})}(t)$ and $U^{(\mathrm{Se})}(t)$.

In the main text, we introduced the sum $U^{(j)}(t) = U_0{}^{(j)} + \Delta U^{(j)}(t)$, in which $U_0{}^{(j)}$ represents the unperturbed MSD of ion j (due to a thermal population of phonons at room temperature), and $\Delta U^{(j)}(t)$ is the time-dependent change in this MSD caused by photoinduced phonons. We can now say that the two $U_0{}^{(j)}$ terms are consistent across *all* delays for *all* reflections, whereas the two temporal terms $\Delta U^{(j)}(t)$ are consistent across all reflections but vary at each delay.

Based on Eq. 1, we define the normalized intensity of each family as $I(t)/I(t<0)$, in which $I(t<0)$ depends only on $U_0{}^{(\mathrm{W})}$ and $U_0{}^{(\mathrm{Se})}$, while $I(t)$ depends also on $\Delta U^{(W)}(t)$ and $\Delta U^{(Se)}(t)$. The experimental values corresponding to this definition are those in Figure 2a of the main text (and in Figure S6a below for clarity).

With these definitions, we performed a linear regression fit on all experimental data shown in Figure S6a, with each dataset therein analytically described by its unique version of Eq. 1. We note that in this procedure all data points simultaneously share ${U_0}^{(Se)}$ and ${U_0}^{(W)}$. This was implemented by extracting the parameters using a nonlinear least-squares solver (in MATLAB, the built-in function "lsqnonlin").

To demonstrate the success of this procedure, a covariance matrix is presented in Figure S5a, in which the correlation between each fit parameter is represented. In the following, we describe this process in detail. Additionally, in Figure S5b, we plot the absolute values of all fit residuals (for every delay and every Bragg reflection). It demonstrates that our fit is in overall good agreement with the data at all delays, and highlights where agreement is lower, most notably for the reflection (2 3 0).

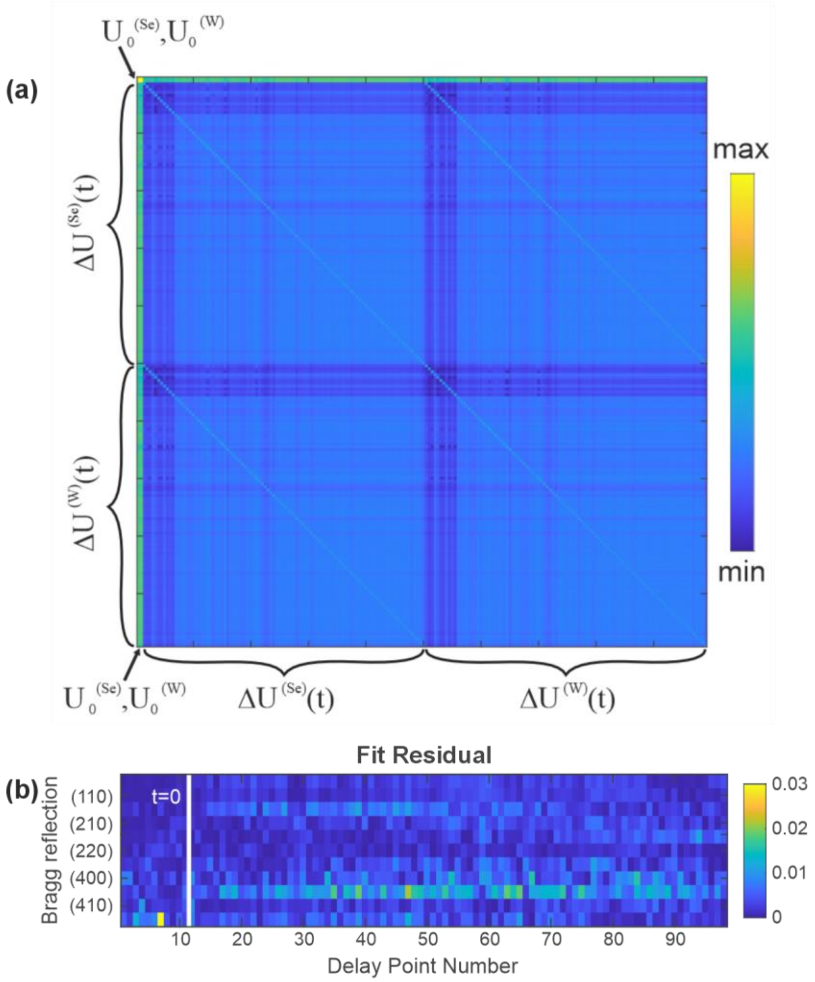

Figure S5 (a) Covariance matrix of the fit parameters in log scale (absolute values). It shows notably strong correlation between the two shared parameters (${U_0}^{(W)}, {U_0}^{(Se)}$) and the other parameters. (the top and the left edges of the matrix) (b) fit residuals at each delay data point for all observed reflections: vertical axis represents the Bragg reflections (by order of q^2) and the horizontal axis represents delay point number. Zero delay is highlighted for reference.

5.2 Signatures of the element-specific MSDs

Here we explain how signatures of the differences between the vibrations of the two atom species can be seen in the raw data. It is common practice in many UED studies to use the so-called "effective-MSD approximation", in which the structure factor description of a Bragg reflections (Eq. 1 and 3 combined) is written as [2,12,13]:

$$I(\boldsymbol{q},t) \propto \left| \sum_j f_j^{(e)}(q)\tau_j(t,\boldsymbol{q}) \exp\left(i\boldsymbol{q}\cdot\boldsymbol{r}_j\right) \right|^2 \approx I_0 \exp(-\langle u^2\rangle q^2) \ .$$

This simplification ignores all differences between MSDs of different sites in the unit cell, and instead focuses only on the overall photoinduced response of the lattice, which can now be written as an *effective* photoinduced MSD, $\langle u^2\rangle \propto -ln\left(\frac{I(t)}{I_0}\right)/q^2$. In this approximation, the normalized intensity $I(t)/I_0$ is a smooth exponential function with respect to q^2 (often appearing as near-linear; note that the q dependence of $I(t)$ and I_0 are omitted for clarity in the expressions above). To demonstrate this, Figure S6b presents the normalized intensities from one delay (21 ps), alongside an exponential function. Qualitative agreement is observed, but deviations from the smooth line are also clearly seen, and they grow with increasing q^2. These deviations are the signatures we are after. They reflect differences in the vibrations between different atoms in the unit cell, and underline the limit of the effective-MSD approximation, which averages out these differences. The same signatures can be seen in recently published UED data about similar systems. [14]

For further insight about these features, Figure S6c presents calculations of normalized intensities for two extreme cases: one in which only W ions are excited and in which only Se ions are excited. This was done using Eq. 1 and 3 in the main text, with $U_0^{(Se)} = 0.006$ Å^2, $U_0^{(W)} = 0.003$ Å^2 (optimal U_0 values obtained, see following), and $\Delta U^{(j)} = 0.004$ Å^2. The two results suggest that each atom species produces markedly different features in the normalized intensity.

5.3 Element-specific MSD fit procedure

In essence, the procedure needs to find the correct ratio between the extreme cases in Figure S6c. The starting parameters for this fit are chosen as follows:

1. The unperturbed (shared) parameters $U_0^{(j)}$ of both elements are set at 0.005 Å^2, based on the thermal MSD values reported for bulk $TiSe_2$ in ref. [15] (~ 0.006 Å^2) and the heavier atomic mass of W.
2. The time-dependent parameters $\Delta U^{(Se)}$ and $\Delta U^{(W)}$ are set at all delays as 0.005 Å^2 and 0.003 Å^2, respectively. This is based on the effective MSD reported for bulk WSe_2 in ref. [13] and considers the difference in atomic masses (W is ~ 2 times heavier than Se).

To ensure that the values remain in a reasonable range, a lower boundary of 0 Å^2 was applied to all parameters, while an upper boundary of 0.01 Å^2 was applied to the shared (unperturbed) parameters $U_0^{(j)}$.

The two-level fit to Eq. 1 is then run, producing results such as the purple line in Figure S6b. The results of these procedures are those presented in Figure 2b of the main text. The covariance matrix of the fit parameters is subsequently calculated. In this matrix, the values on the diagonal represent the variances of each parameter (which is related to standard deviations) and the off-diagonal values are the covariances (this reflects correlation) between pairs of parameters. The matrix is depicted in Figure S5, where higher values indicate stronger correlation between the two parameters (note that the color scale is logarithmic). Figure S5d shows a notably high correlation in between the two unperturbed parameters $U_0^{(Se)}$ and $U_0^{(W)}$, as well as between them and the $\Delta U^{(j)}$ parameters at each delay. This strong correlation demonstrates the successful sharing of parameters $U_0^{(Se)}$ and $U_0^{(W)}$ during the fit process. However, this increased correlation might contribute to the large uncertainties of the shared parameters. We note here that the procedure produces the unperturbed MSDs ($U_0^{(Se)} = 0.006$ Å^2 and $U_0^{(W)} = 0.003$ Å^2) with undetermined standard errors. We address this in the next section.

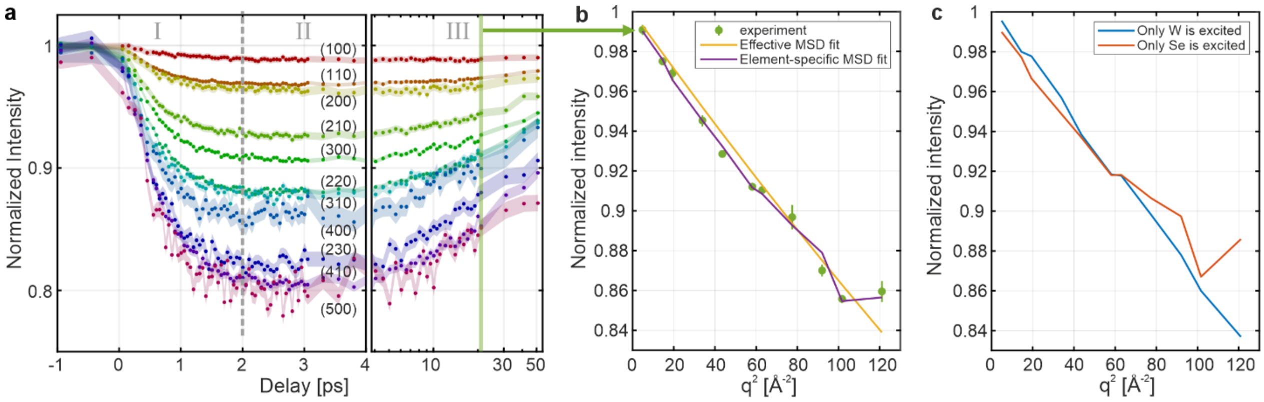

Figure S6 (a) The same panel as in Figure 2a in the main text. (b) Example of agreement between the data and the global fit: experimental intensity at a delay of 21 ps as function of q^2, alongside the intensity curves obtained by the effective MSD fit and the global fit at this delay, respectively (c) Calculated normalized intensity from two cases: the blue line is the calculated intensity when only W ions are excited ($\Delta U^{(W)} = 0.004$ Å^2, $\Delta U^{(Se)} = 0$ Å^2) and the red line is when only Se ions are excited ($\Delta U^{(W)} = 0$ Å^2, $\Delta U^{(Se)} = 0.004$Å^2). The values used for $U_0^{(W)}$ and $U_0^{(Se)}$ are the same as in the fit result in (b). The unique fluctuations of the two lines imply distinctive element-specific contribution in the structure factor.

5.4 The limit of sensitivity to U_0

Despite of the good agreement between the extracted $U_0^{(j)}$ values and the literature as shown in the main text, the undetermined confidence intervals for the U_0 values are a weakness of this approach. Such values are normally determined through crystal structure refinement of diffraction patterns.

As this casts doubt on our findings regarding the $\Delta U^{(j)}$ dynamics, we investigated this by systematically repeating the fit routine with many different fixed $U_0^{(j)}$ values. For each fixed fit, we evaluated the sum of least squares. This is presented as a color map in Figure S7(a), revealing a clear preference of $U_0^{(Se)} > U_0^{(W)}$. A thick red line is indicated, along which we observe local minima that fulfill the relation $U_0^{(Se)} \approx U_0^{(W)} + 0.003$ Å^2.

To understand this, we investigated the q^2-dependence of normalized intensity generated by these minima (as in Figure S6c). We find no difference in the $\Delta U^{(j)}$ dynamics generated by fits along this line. To demonstrate this, Figure S7b presents data comparable to that in Fig S2c generated from fits at two such minima: $U_0^{(Se)}$=0.010, $U_0^{(W)}$=0.007, and $U_0^{(Se)}$=0.004, $U_0^{(W)}$=0.001 (all in units of Å^2). The difference between the curves is at the computational limit, so we conclude that the undetermined confidence intervals for the $U_0^{(j)}$ values stem from the fits' insensitivity to $U_0^{(j)}$ values along the $U_0^{(Se)} \approx U_0^{(W)} + 0.003$ Å^2 line, but that this relation should be maintained. For a comparison, Figure S7c presents the fit at the maximum of the color map in Figure S7a ($U_0^{(Se)}$=0.0065 Å^2, $U_0^{(W)}$=0.0085 Å^2). The fit result does not reveal any clear trend in the MSDs and produces larger uncertainties.

5.5 Comparison with unperturbed MSD calculated in the study of I.-C. Tung et al.

In the supplementary information of ref. [16] unperturbed in-plane MSD values were calculated for monolayer WSe_2 using DFT: 0.004 Å^2 and 0.003 Å^2 for Se and W ions, respectively. These values deviate from ours. These DFT values are $\langle u^2 \rangle_{xy}$, which is defined along either x or y in cartesian coordinates. In contrast, we used the elements $U_{11}^{(j)} = U_{22}^{(j)} = 2U_{12}^{(j)} \equiv U^{(j)}$ from the definition of a U-matrix in Ref. [9], where each element points along the crystal axes, such that 11 and 22 are not orthogonal in the symmetry of WSe_2. Nonetheless, as the in-plane dynamics in the crystal are isotropic, the values from U-matrix and those along the cartesian axes are identical in-plane; $U^{(j)} = \langle u^2 \rangle_{xy}$. (This can be proven by the conversion matrix in Ref. [9])

The position of these values is indicated in Figure S7a. We fixed these values and conducted the same fit routine, which resulted in the dynamics shown in Figure S7d, and the q^2 dependence at 21 ps in Figure S7e. We find qualitative agreement with the trends observed in the main text (Figure 2b), albeit with larger error bars and clear disagreement in the q^2 dependence.

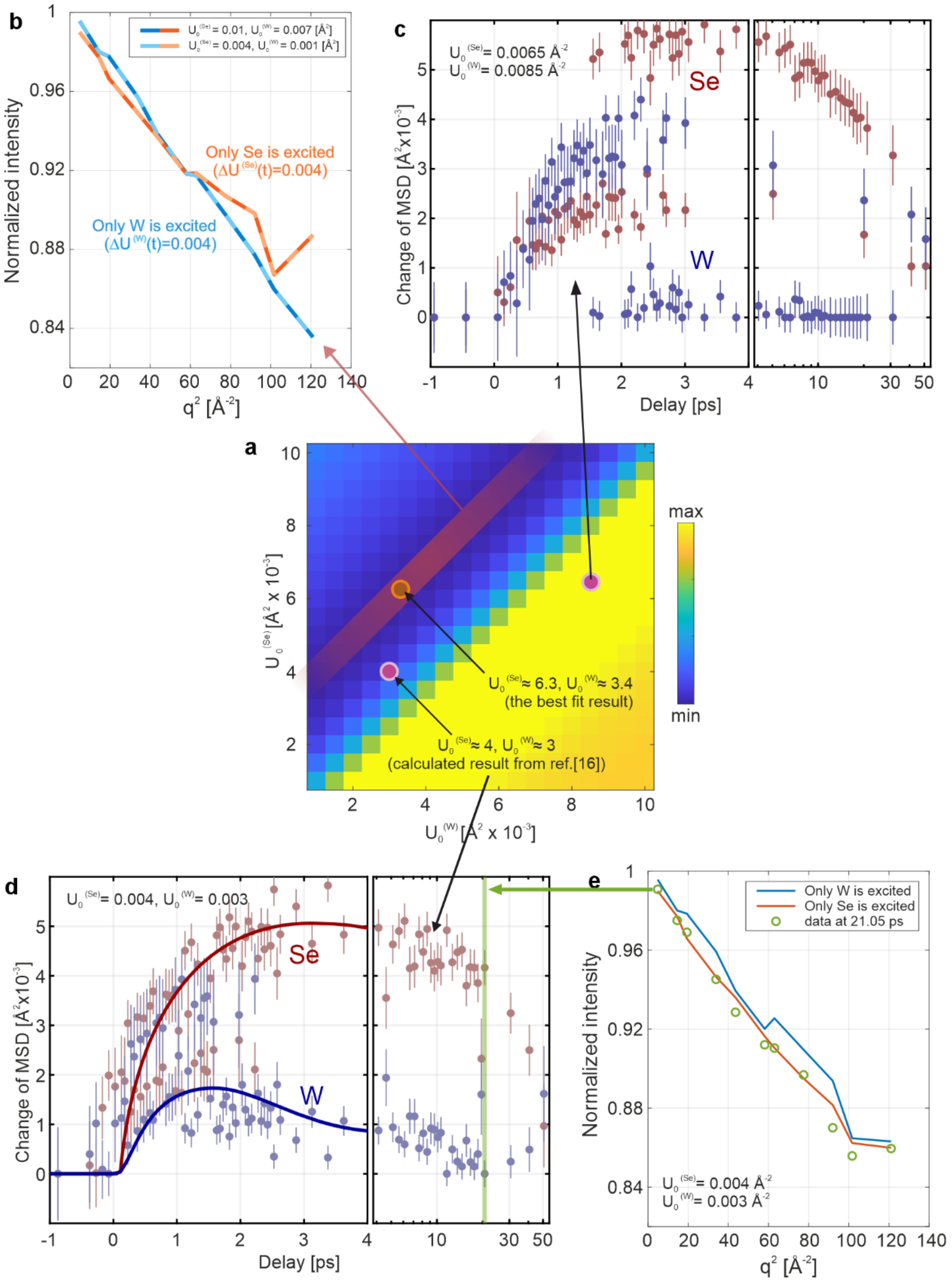

Figure S7 (a) A map of the sum of least squares fits with $U^{(j)}$ values fixed. Optimal conditions fulfill $U_0^{(\mathrm{W})} < U_0^{(\mathrm{Se})}$, and a series of local minima are observed along the thick red line. The result from the initial fit (without fixed values) is on this line. Values from Ref. [16] are indicated (b) Similar calculation to Figure S6c, but using two pairs of $U_0^{(\mathrm{W})}$ and $U_0^{(\mathrm{Se})}$ values; $U_0^{(\mathrm{Se})} = 0.01$, $U_0^{(\mathrm{W})} = 0.007$, and $U_0^{(\mathrm{Se})} = 0.004$, $U_0^{(\mathrm{W})} = 0.001$ Å^2. Both cases are plotted as dashed lines on top of each other. (c) Element-specific MSDs using fixed unperturbed fit parameters at a maximum of the map in (a) ($U_0^{(\mathrm{Se})} = 0.0065$ Å^2 and $U_0^{(\mathrm{W})} = 0.0085$ Å^2). (d) Change of MSDs from a fit in which the U_0 values from ref. [16] were used. (e) similar calculation to Figure S6c, corresponding to the fit in (d).

6 Comparison to previously reported monolayer WSe_2 data

Ref. [16] reported pump-probe X-ray diffraction data taken from monolayer WSe_2 in reflection geometry. As these results relate to ours, we discuss them here. First, we demonstrate agreement with our data by assessing the time dependence of intensity attenuation due to the Debye-Waller effect. For close comparison with the phenomenological exponential fits to intensity of ref. [16], we use the averaged normalized intensity of the Bragg reflections of the (100) family in a delay window of up to 10 ps (Figure S6a). These data were fit to a function of the form $A\exp(-\Delta t/\tau)$ convoluted with a Heaviside function and a gaussian resolution function with a width of 0.2 ps. (Figure S8) Here A is the relative intensity drop, Δt is the time delay, and τ denotes the relaxation time constant. This analysis produced $\tau = 0.75 \pm 0.06$ ps, in very good agreement with the "in-plane electron–phonon coupling time" 0.8 ± 0.4 ps reported in ref. [16].

However, the conditions in the two studies are different, casting doubt on the validity of comparison between the two studies. In ref. [16], the pump photon energy was resonant to the B-exciton (650 nm), producing a carrier density of 1.3×10^{13} cm^{-2}. This is approximately 35 times lower than the carrier density in our work, and is slightly above the excitonic Mott transition density for bulk (~8×10^{12} cm^{-2}). [17]. Given that in monolayer TMDCs the excitonic binding energy is stronger than in bulk materials [18,19], higher critical densities for the Mott transition can be expected. As such, the dynamics in ref. [16] may reflect lattice dynamics resulting from excitonic effects, which may differ from the quasi-free particle picture in our work [20]. In particular, B excitons are reported to mainly couple with LA phonons [20], while quasi-free carriers couple with several higher energy modes. [13] For these reasons, direct comparison between the two studies is not sound.

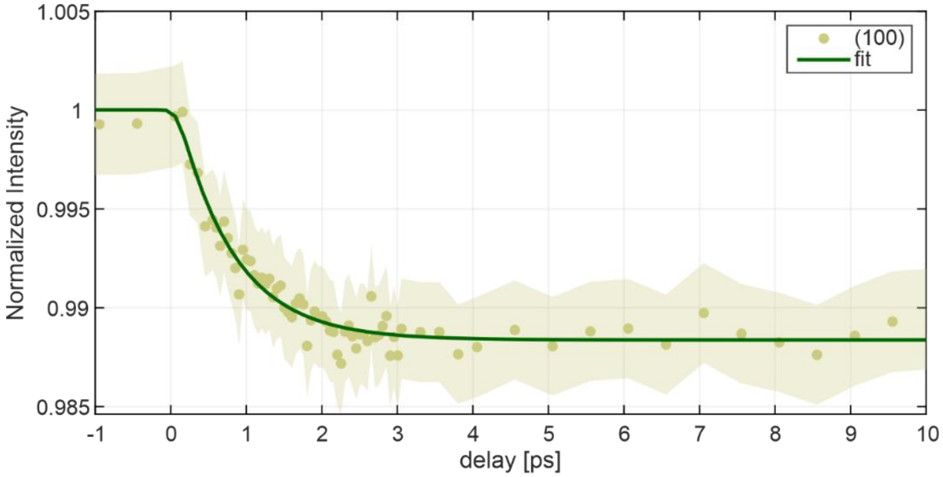

Figure S8 Average of the normalized intensities from the Bragg reflection family (100), overlayed with the best fit to an exponential decay function. The shaded area represents the certainty of the data points, taken as their standard deviation.

7 References

[1] R. Schmidt, I. Niehues, R. Schneider, M. Drüppel, T. Deilmann, M. Rohlfing, S. M. de Vasconcellos, A. Castellanos-Gomez, and R. Bratschitsch, *Reversible Uniaxial Strain Tuning in Atomically Thin* WSe_2, 2d Mater **3**, 021011 (2016).

[2] A. Sood, J. B. Haber, J. Carlström, E. A. Peterson, E. Barre, J. D. Georgaras, A. H. M. Reid, X. Shen, M. E. Zajac, E. C. Regan, et al., *Bidirectional Phonon Emission in Two-Dimensional Heterostructures Triggered by Ultrafast Charge Transfer*, Nat Nanotechnol **18**, 29 (2023).

[3] H. Gu, B. Song, M. Fang, Y. Hong, X. Chen, H. Jiang, W. Ren, and S. Liu, *Layer-Dependent Dielectric and Optical Properties of Centimeter-Scale 2D* WSe_2*: Evolution from a Single Layer to Few Layers,* **11**, (2019).

[4] *M. N. Polyanskiy, 10 10 2021. [Online]. Available: Https://Refractiveindex.Info.*, (unpublished).

[5] H. Seiler, D. Zahn, M. Zacharias, P.-N. Hildebrandt, T. Vasileiadis, Y. W. Windsor, Y. Qi, C. Carbogno, C. Draxl, R. Ernstorfer, et al., *Accessing the Anisotropic Nonthermal Phonon Populations in Black Phosphorus*, Nano Lett **21**, 6171 (2021).

[6] M. Born and E. Wolf, *Principles of Optics* (Pergamon Press, Oxford, 1964).

[7] W. J. Schutte, J. L. De Boer, and F. Jellinek, *Crystal Structures of Tungsten Disulfide and Diselenide,* J Solid State Chem **70**, 207 (1987).

[8] R. Mincigrucci, D. Naumenko, L. Foglia, I. Nikolov, E. Pedersoli, E. Principi, A. Simoncig, M. Kiskinova, C. Masciovecchio, F. Bencivenga, et al., *Optical Constants Modelling in Silicon Nitride Membrane Transiently Excited by EUV Radiation*, Opt Express **26**, 11877 (2018).

[9] B. T. M. Willis and A. W. Pryor, *Thermal Vibrations in Crystallography*, 1st ed. (Cambridge University Press, 1975).

[10] L. M. Peng, S. L. Dudarev, and M. J. Whelan, *High Energy Electron Diffraction and Microscopy* (Oxford University Press, Oxford, 2004).

[11] R. Xu and T. C. Chiang, *Determination of Phonon Dispersion Relations by X-Ray Thermal Diffuse Scattering*, Zeitschrift Für Kristallographie **220**, 1009 (2005).

[12] M.-F. Lin, V. Kochat, A. Krishnamoorthy, L. Bassman Oftelie, C. Weninger, Q. Zheng, X. Zhang, A. Apte, C. S. Tiwary, X. Shen, et al., *Ultrafast Non-Radiative Dynamics of Atomically Thin* $MoSe_2$, Nat Commun **8**, 1745 (2017).

[13] L. Waldecker, R. Bertoni, H. Hübener, T. Brumme, T. Vasileiadis, D. Zahn, A. Rubio, and R. Ernstorfer, *Momentum-Resolved View of Electron-Phonon Coupling in Multilayer* WSe_2, Phys Rev Lett **119**, 036803 (2017).

[14] A. C. Johnson, J. D. Georgaras, X. Shen, H. Yao, A. P. Saunders, H. J. Zeng, H. Kim, A. Sood, T. F. Heinz, A. M. Lindenberg, et al., *Hidden Phonon Highways Promote Photoinduced Interlayer*

Energy Transfer in Twisted Transition Metal Dichalcogenide Heterostructures, Sci Adv **10**, (2024).

[15] C. Riekel, *Structure Refinement of $TiSe_2$ by Neutron Diffraction*, J Solid State Chem **17**, 389 (1976).

[16] I.-C. Tung, A. Krishnamoorthy, S. Sadasivam, H. Zhou, Q. Zhang, K. L. Seyler, G. Clark, E. M. Mannebach, C. Nyby, F. Ernst, et al., *Anisotropic Structural Dynamics of Monolayer Crystals Revealed by Femtosecond Surface X-Ray Scattering*, Nat Photonics **13**, 425 (2019).

[17] A. Steinhoff, M. Florian, M. Rösner, G. Schönhoff, T. O. Wehling, and F. Jahnke, *Exciton Fission in Monolayer Transition Metal Dichalcogenide Semiconductors*, Nat Commun **8**, 1166 (2017).

[18] M. M. Ugeda, A. J. Bradley, S.-F. Shi, F. H. da Jornada, Y. Zhang, D. Y. Qiu, W. Ruan, S.-K. Mo, Z. Hussain, Z.-X. Shen, et al., *Giant Bandgap Renormalization and Excitonic Effects in a Monolayer Transition Metal Dichalcogenide Semiconductor*, Nat Mater **13**, 1091 (2014).

[19] A. Chernikov, T. C. Berkelbach, H. M. Hill, A. Rigosi, Y. Li, O. B. Aslan, D. R. Reichman, M. S. Hybertsen, and T. F. Heinz, *Exciton Binding Energy and Nonhydrogenic Rydberg Series in Monolayer WS_2*, Phys Rev Lett **113**, 076802 (2014).

[20] H. Mishra, A. Bose, A. Dhar, and S. Bhattacharya, *Exciton-Phonon Coupling and Band-Gap Renormalization in Monolayer WSe_2*, Phys Rev B **98**, 045143 (2018).
